# New York State Veterinary Medical Society

**Published:** 1900-07

**Authors:** 


### NEW YORK STATE VETERINARY MEDICAL SOCIETY.

President Bell, of the New York State Veterinary Medical Society, states that strenuous efforts are being put forth by the officers of the Society to make the forthcoming meeting, to be held at Ithaca the week following that of the American Association at Detroit, one of great interest and value to veterinarians, and that a cordial invitation is extended to the members of the profession of sister States to join with them on this occasion. There will undoubtedly be a feast of reason, as papers are promised from members throughout the State upon subjects of great interest and importance, while the clinics will include surgical demonstrations of the most varied and practical character. The Committee of Arrangements are preparing a programme of delightful social diversions, and it is hoped that all who can will avail themselves of the cordial invitation extended.

